# Comparative genomic and functional analysis of *Akkermansia muciniphila* and closely related species

**DOI:** 10.1007/s13258-019-00855-1

**Published:** 2019-08-09

**Authors:** Juyuan Xing, Xiaobo Li, Yingjiao Sun, Juanjuan Zhao, Shaohua Miao, Qin Xiong, Yonggang Zhang, Guishan Zhang

**Affiliations:** 1 Key Laboratory of Microbial Resources Collection and Preservation, Ministry of Agriculture, Institute of Agricultural Resources and Regional Planning, Chinese Academy of Agricultural Sciences, No. 12 Zhongguancun South Street, Haidian District, Beijing, 100081 People’s Republic of China; 2 Wuhan University of Technology, Wuhan, Hubei People’s Republic of China; 3 BGI Education Center, University of Chinese Academy of Sciences, Shenzhen, China; 4 Biology Institute, Qilu University of Technology (Shandong Academy of Sciences), No. 19 Keyuan Road, Jinan, 250014 Shandong People’s Republic of China; 5 BGI-Shenzhen, Shenzhen, China; 6 China National GeneBank-Shenzhen, BGI-Shenzhen, Shenzhen, China

**Keywords:** *Akkermansia muciniphila*, Comparative genome, Phylogenetic analysis, Pan-genome, Gene clusters

## Abstract

**Background:**

*Akkermansia muciniphila* is an important bacterium that resides on the mucus layer of the intestinal tract. *Akkermansia muciniphila* has a high abundance in human feces and plays an important role in human health.

**Objective:**

In this article, 23 whole genome sequences of the *Akkermansia* genus were comparatively studied.

**Methods:**

Phylogenetic trees were constructed with three methods: All amino acid sequences of each strain were used to construct the first phylogenetic tree using the web server of Composition Vector Tree Version 3. The matrix of Genome-to-Genome Distances which were obtained from GGDC 2.0 was used to construct the second phylogenetic tree using FastME. The concatenated single-copy core gene-based phylogenetic tree was generated through MEGA. The single-copy genes were obtained using OrthoMCL. Population structure was assessed by STRUCTURE 2.3.4 using the SNPs in core genes. PROKKA and Roary were used to do pan-genome analyses. The biosynthetic gene clusters were predicted using antiSMASH 4.0. IalandViewer 4 was used to detect the genomic islands.

**Results:**

The results of comparative genomic analysis revealed that: (1) The 23 *Akkermansia* strains formed 4 clades in phylogenetic trees. The *A. muciniphila* strains isolated from different geographic regions and ecological niches, formed a closely related clade. (2) The 23 *Akkermansia* strains were divided into 4 species based on digital DNA-DNA hybridization (dDDH) values. (3) Pan-genome of *A. muciniphila* is in an open state and increases with addition of new sequenced genomes. (4) SNPs were not evenly distributed throughout the *A. muciniphila* genomes. The genes in regions with high SNP density are related to metabolism and cell wall/membrane envelope biogenesis. (5) The thermostable outer-membrane protein, Amuc_1100, was conserved in the *Akkermansia* genus, except for *Akkermansia glycaniphila* Pyt^T^.

**Conclusion:**

Overall, applying comparative genomic and pan-genomic analyses, we classified and illuminated the phylogenetic relationship of the 23 *Akkermansia* strains. Insights of the evolutionary, population structure, gene clusters and genome islands of *Akkermansia* provided more information about the possible physiological and probiotic mechanisms of the *Akkermansia* strains, and gave some instructions for the in-depth researches about the use of *Akkermansia* as a gut probiotic in the future.

**Electronic supplementary material:**

The online version of this article (10.1007/s13258-019-00855-1) contains supplementary material, which is available to authorized users.

## Introduction

*Akkermansia muciniphila,* a new member of the *Verrucomicrobia* phylum (Hedlund et al. 1997), was first isolated from human feces as a new mucus-degrading bacterium in 2004 (Derrien et al. 2004). *A. muciniphila,* which is an anaerobic Gram-negative bacterium commonly found within the mucus layer of the digestive tract, is isolated by anaerobic medium containing gastric mucin as the sole carbon and nitrogen source (Derrien et al. 2004). Thus far, there are two species in the *Akkermansia* genus as follows: *A. muciniphila* ATCC BAA-835^T^, which was first isolated from human feces in 2004 (Derrien et al. 2004); and *Akkermansia glycaniphila* Pyt^T^, which was first isolated from reticulated python feces in 2016 (Ouwerkerk et al. 2016). Metagenome data suggest that there are at least eight different species of the *Akkermansia* genus in the digestive tract of humans apart from *A. muciniphila*, and different species exist in the same region simultaneously (van Passel et al. 2011). The differences of ecological niches and hosts of *Akkermansia* are tremendous. *Akkermansia*-like organisms are found in the intestines of other non-human mammals, such as lemur, gorilla (Ley et al. 2008) and mice (Presley et al. 2010), as well as other vertebrates, such as chickens (Belzer and de Vos 2012) and zebrafish (Roeselers et al. 2011). Moreover, 16S rRNA sequences of the *Akkermansia* genus are universally detected in animals, ranging from domesticated and wild mammals to non-mammals, such as birds and fish (Belzer and de Vos 2012), as well as to reptiles, such as the Burmese python (Costello et al. 2010). The gastrointestinal tract anatomy, diet and mucin types of these animals are largely different.

The first available *A. muciniphila* genome sequence was for the strain ATCC BAA-835^T^, which was sequenced in 2011 (van Passel et al. 2011). The whole genome of this strain is composed of one circular chromosome of 2.66 Mbp with an average G + C content of 55.8%. The *A. glycaniphila* Pyt^T^ genome was sequenced in 2017 (Ouwerkerk et al. 2017), comprising one circular chromosome of 3.07 Mbp with a G + C content of 57.6%. Compared with other genomes of the *Verrucomicrobia* phylum, the *A. muciniphila* genome showed distinct phylogenetic features as only 28.8% of genes were shared with its closest relative (van Passel et al. 2011). At present, whole-genome assembly of *A. muciniphila* can be sequenced directly from human stool in the absence of a cultured isolate (Caputo et al. 2015).

*Akkermansia muciniphila* plays an important role in maintaining a healthy mucus layer in the human gut (Derrien et al. 2017), and it may represent 3–5% of the microbial composition in the healthy human intestinal tract (Derrien et al. 2004; Belzer and de Vos 2012). Studies have identified a loss in abundance of *A. muciniphila* in patients with obesity and type 2 diabetes (T2D) (Derrien et al. 2011; Everard et al. 2013). Moreover, increasing evidence has shown that the abundance of *A. muciniphila* is related with body weight, type 1 diabetes (T1D) (Hansen et al. 2012), inflammatory bowel disease (Png et al. 2010), autism (Wang et al. 2011) and cancer (Weir et al. 2013). The analysis of *A. muciniphila* genome predicts that over 61 (11%) of proteins are involved in the degradation of mucin (Belzer and de Vos 2012). However, the precise physiological mechanisms affected by this bacterium during metabolic disorders and intestinal permeability regulation remain unclear, and much of the *Akkermansia* genus lacks in-depth analysis and comparative features among *Akkermansia* strains.

In the present study, 23 whole genome sequences of the *Akkermansia* genus were selected from NCBI databases for comparative studies. Digital DNA-DNA hybridization (dDDH) values suggested that these whole genome sequences came from different species of the *Akkermansia* genus. Phylogenetic analysis of the concatenated single-copy core genes suggested that 18 whole genome sequences formed a closely related clade. Pan-genome analyses were compared among these 18 genome sequences. Analysis of gene clusters and genomic islands suggested that these gene sequences were not conserved and that they may come from other species by lateral gene transfer (LGT).

## Materials and methods

### Characterization of *Akkermansia* strains and closely related species

Twenty-three strains of the *Akkermansia* genus were selected for comparative genome analysis from NCBI. The other 4 strains from the *Verrucomicrobia* phylum were selected based on species that were closely related to the *Akkermansia* genus. The genome sequences and annotation information for the strains were downloaded from NCBI databases.

### Genomic quality assessment

The completeness and contamination of the genome sequences which recovered from metagenomes were evaluated by the software CheckM (Parks et al. 2015).

### dDDH values

The results of the genome comparisons for taxonomic purpose were collected using BLAST2.7.1 + (Camacho et al. 2009). The digital DNA-DNA hybridization (dDDH) values were obtained by means of genome-to-genome sequence comparison via GGDC 2.0 using Formula 1 (Meier-Kolthoff et al. 2013), and the digital DNA-DNA hybridization (dDDH) was used to replace the tedious traditional approach (Auch et al. 2010).

### Phylogenetic analysis

Phylogenetic trees were constructed with the following three methods: two trees constructed using whole-genome-based methods; and the other tree was constructed using the concatenated single-copy core gene sequences. All amino acid sequences of each strain were used to construct the first phylogenetic tree using the web server of Composition Vector Tree Version 3 (CVTree3), and 6 was the K-tuple length (Zuo and Hao 2015). All dDDH values obtained from GGDC 2.0 were based on Genome-to-Genome Distances (GGDs) calculated by the Genome BLAST Distance Phylogeny (GBDP) approach (Henz et al. 2005; Auch et al. 2006), and the matrix of these GGDs was used to infer the second phylogenetic tree with FastME (Desper and Gascuel 2002). The concatenated single-copy core gene-based phylogenetic tree was generated through MEGA (Kumar et al. 2016) using the Neighbor Joining (NJ) method with 1000 bootstrap replicates, and *Rubritalea marina* DSM 18772^T^ was the out-group. OrthoMCL was used to obtain orthologous genes of the strains to construct a phylogenetic tree (Li et al. 2003). The single-copy genes were obtained from the OrthoMCL results, and the concatenated single-copy core gene sequences were aligned using MAFFT (Katoh et al. 2017).

### Population structure

Population structure was assessed by STRUCTURE 2.3.4 (Evanno et al. 2005) using the SNPs in core genes shared by 23 *Akkermansia* genomes. Of note, *K* was varied from 1 to 8 with a burn-in of 5000 iterations. The best number of populations (*K*) was identified using δ*K* via the method of Evanno et al. (2005).

### Variant calls: SNPs

Firstly, single nucleotide polymorphism (SNP) calls were performed using Snippy, which uses BWA Mem (Li and Durbin 2009) to map the 250 bp single-end reads that were shredded from the contigs to the reference *A. muciniphila* ATCC BAA-835^T^ and then calls the SNPs with FreeBayes (Garrison and Marth 2012). Whole genome alignment output from Snippy were used to identified the final result about SNPs distribution of the 18 *A. muciniphila* strains, using Gubbins (Genealogies Unbiased By recombinations In Nucleotide Sequences) (Croucher et al. 2015). The density of SNP distribution was calculated throughout the genome sequence using a sliding-window size of 5 kb (step of the sliding window = 5 kb).

### Pan-genome analyses of *Akkermansia muciniphila*

Roary is a high-speed pan genome pipeline (Page et al. 2015), which takes annotated assemblies in the GFF3 format and calculates the pan-genome. The GFF3 format files of the 18 genomes of *A. muciniphila* were produced using PROKKA (Seemann 2014). The gene accumulation curve was produced via ggplot2 using the results of Roary. A Venn diagram was used to show the core genes and strain-specifics of the 18 *A. muciniphila* strains.

### Identification of functional categories for core and strain-specific genes

To identify the functional categories of core and strain-specific genes of the 18 *A. muciniphila* strains, Clusters of Orthologous Groups (COGs) databases were used (Tatusov et al. 2003).

### Analyses of gene clusters and genomic islands

The biosynthetic gene clusters of ATCC BAA-835^T^ strain were predicted using antiSMASH 4.0 (Blin et al. 2017). IslandViewer 4 was used to detect the genomic islands of ATCC BAA-835^T^ strain (Bertelli et al. 2017). The corresponding orthologous genes of the gene clusters and genomic islands in other strains were identified using OrthoMCL. All protein sequences were compared using BLASTP all-against-all with an E value cutoff of 1e-05 and 70% similarity. The Markov Cluster (MCL) algorithm was used to determine orthologous groups using a value of 2.0 (Enright et al. 2002). The synteny maps of the gene clusters and genomic islands were generated using R package genoPlotR (Guy et al. 2011).

## Results

### Genomic features and genomic quality assessment

A summary of the features of each of the 23 genomes of the *Akkermansia* genus and 4 genomes of its closely related species is shown in Table 1. The G + C contents of the 23 genomes ranged from 55 to 58.1%. Their genome sizes varied from 2.43 to 3.11 Mb with the number of CDSs ranging from 2045 to 2526. Compared with the other 4 genomes from the *Verrucomicrobia* phylum, the genomes of the *Akkermansia* genus were much smaller in size and had fewer CDSs, indicating the large difference between *Akkermansia* with other species of the *Verrucomicrobia* phylum. The genome sequences of the *Akkermansia* strains Urmite, CAG:154, CAG:344, UNK.MGS-1, 54_64, Phil8, UBA3271, UBA7059 and UBA7090 were recovered from metagenomes, so the genome may have sequence contamination. The software CheckM (Parks et al. 2015) was used to evaluate the genomes quality of these strains. The completeness and contamination of the sequences was listed in Table 1. The results suggested that these sequences have high quality with high sequence completeness and less contamination.

**Table 1:** Features and genomic quality of all the genomes studied in this article

Feature	Genome size (bp)	G + C (%)	Scaffolds	Genes	CRISPR	Source	Accession number	Metagenome bin	Completeness (%)	Contamination (%)
*Akkermansia* *muciniphila* ATCC BAA-835^T^	2,664,102	55.8	1	2321	2	Human feces	GCA_000020225.1	No		
*Akkermansia* *glycaniphila* APytT^T^	3,074,078	57.6	1	2575	4	APytT	GCA_900097105.1	No		
*Akkermansia* *muciniphila* YL44	2,745,278	55.7	1	2413	2	Mouse	GCA_001688765.2	No		
*Akkermansia* *muciniphila* Urmite	2,664,738	55.7	1	2307	0	Human stool	GCA_000723745.2	Yes	96.599	0
*Akkermansia* sp. KLE1797	3,109,783	56.9	51	2642	8	Human feces	GCA_001578645.1	No		
*Akkermansia* sp. KLE1798	3,113,726	56.9	40	2636	11	Human feces	GCA_001580195.1	No		
*Akkermansia* sp. KLE1605	3,108,219	56.9	54	2632	12	Human GI tract	GCA_001647615.1	No		
*Akkermansia* *muciniphila* An78	2,736,972	55.7	10	2385	1	Chicken caecum	GCA_002161325.1	No		
*Akkermansia* *muciniphila* (Horse)	2,774,487	55.7	90	2438	2	Horse	GCA_900184855.1	No		
*Akkermansia muciniphila* (Siamang)	2,652,501	55.8	110	2333	2	Siamang	GCA_900184905.1	No		
*Akkermansia muciniphila* (Mouse)	2,715,564	55.6	130	2460	2	Mouse	GCA_900184985.1	No		
*Akkermansia muciniphila* (Pig)	2,807,935	55.8	70	2456	2	Pig	GCA_900185065.1	No		
*Akkermansia muciniphila* (Chimpanzee)	2,615,504	55.7	77	2298	2	Chimpanzee	GCA_900184845.1	No		
*Akkermansia muciniphila* (Reindeer)	2,672,402	55.8	104	2350	2	Reindeer	GCA_900185005.1	No		
*Akkermansia muciniphila* (Elephant)	2,674,166	55.7	188	2364	2	Elephant	GCA_900185045.1	No		
*Akkermansia* *muciniphila* CAG:154	2,721,828	55.5	81	2431	1	Human gut metagenome	GCA_000436395.1	Yes	97.072	0
*Akkermansia* sp. CAG:344	3,024,253	58.1	78	2577	3	Human gut metagenome	GCA_000437075.1	Yes	97.249	1.361
*Akkermansia* sp. UNK.MGS-1	2,770,805	55	40	2487	2	Human stool samples	GCA_000980515.1	Yes	92.063	3.401
*Akkermansia* sp. 54_46	2,966,498	55.3	36	2586	4	Human gut metagenome	GCA_001917295.1	Yes	97.279	0
*Akkermansia* sp. Phil8	2,437,735	55.3	37	2126	3	Phil the wombat	GCA_001940945.1	Yes	88.435	0.68
*Akkermansia* sp. UBA3271	2,450,463	56	37	2127	4	Rat gut metagenome	GCA_002362435.1	Yes	95.872	0.68
*Akkermansia* sp. UBA7059	2,439,829	56.1	31	2103	2	Rat gut metagenome	GCA_002492685.1	Yes	97.612	0
*Akkermansia* sp. UBA7090	2,447,227	56.2	31	2102	2	Rat gut metagenome	GCA_002493465.1	Yes	97.732	0
*Rubritalea squalenifaciens* DSM 18772^T^	4,322,488	52	17	3884	0	Marine sponge	GCA_900141815.1	No		
*Rubritalea marina* DSM 17716^T^	3,013,271	51.6	30	2650	0	Mediterranean sponge	GCA_000378105.1	No		
*Verrucomicrobium spinosum* DSM 4136^T^	8,220,857	60.3	1	6420	2	Soil	GCA_000172155.1	No		
*Terrimicrobium sacchariphilum* NM-5^T^	4,751,807	60.2	3	4204	0	Rice-field soil	GCA_001613545.1	No		

### dDDH values

The matrix of digital DNA-DNA hybridization (dDDH) values of genome-to-genome is shown in Table S1. ANI values in the range of 95–96% (Richter and Rossello-Mora 2009) correspond to a 70% DDH standard for species definition (Wayne et al. 1987; Rosello-Mora and Amann 2001). Based on the species standard mentioned above, the 23 strains of the *Akkermansia* genus were divided into 4 species as follows: CAG:344 strain, a novel species of the *Akkermansia* genus; KLE1605 strain, KLE1797 strain and KLE1798 strain, which should be identified as another novel species of the *Akkermansia* genus. The two novel species were different from the published *A. muciniphila* and *A. glycaniphila* species, which was confirmed further by the following phylogenetic analysis.

### Phylogenetic analyses

Two whole-genome-based methods were used to construct phylogenetic trees. *Rubritalea marina* DSM 17716^T^, *Rubritalea aqualenifaciens* DSM 18772^T^, *Terrimicrobium sacchariphilum* NM-5^T^ and *Verrucomicrobium spinosum* DSM 4136^T^ were selected as out-groups (Fig S1 and Fig S2) (Kasai et al. 2007; Scheuermayer et al. 2006; Qiu et al. 2014). The first method was alignment-free using whole amino acid sequences, and the second method depended on the distance matrix of all genome-to-genome comparisons (Table S2). The two phylogenetic trees were similar with 18 genome sequences of *A. muciniphila* clustered to one clade of the phylogenetic tree. The dDDH values of 17 other strains of this clade with *A. muciniphila* ATCC BAA-835^T^ ranged from 75.5 to 99.9%, which revealed that the 18 strains from the digestive tracts of human being, mouse, chimpanzee, elephant, horse, siamang, pig, reindeer, chicken and other animals can survive in diverse habitats, suggesting that *Akkermansia* has broad host adaptation. The genome sequences of *A. muciniphila* in different mammals were exactly similar with each other. The strains YL44 and Mouse isolated from mouse have close phylogenetic relation. And the strains UBA3271, UBA7090 and UBA7059 isolated from rat gut are in the same branch of the phylogenetic tree.

**Fig. 1:**
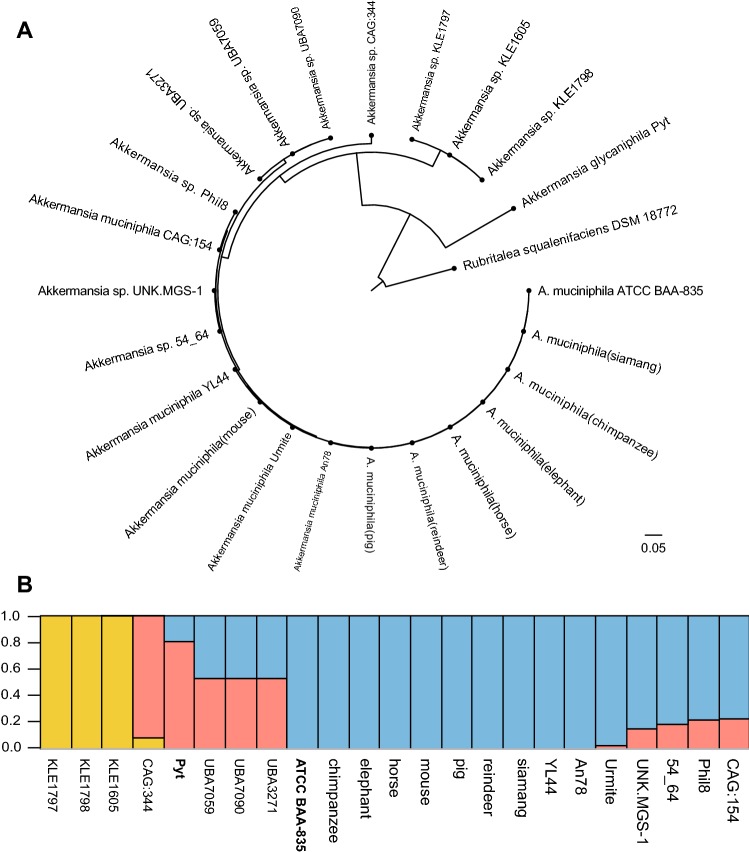
Phylogenetic relationship of 23 *Akkermansia* strains and population structure of 18 *Akkermansia muciniphila* strains. **a** Neighbor Joining (NJ) phylogenetic tree was constructed using the concatenated nucleotide sequences of 710 single-copy core genes shared by 23 *Akkermansia* genomes and an out-group (*Rubritalea squalenifaciens* DSM 18772^T^). The phylogenetic tree was rooted by the out-group. **b** The 18 strains were divided into 3 populations (*K *= 3), and individuals are shown by thin vertical lines, which are divided into *K* colored segments representing the estimated membership probabilities (Q) of each individual (color figure online)

Concatenated core gene alignments are frequently used for constructing a genome-based phylogenetic tree. A total of 710 single-copy core genes were identified by comparison of 23 *Akkermansia* genomes and *Rubritalea aqualenifaciens* DSM 18772^T^. The phylogenetic tree of the 23 *Akkermansia* genomes was constructed based on the concatenation of the 710 core genes that were present in single-copy in all genomes using the Neighbor-joining (NJ) method with 1000 bootstrap replicates (Fig. 1a) and rooted by *Rubritalea aqualenifaciens* DSM 18772^T^. This phylogenetic tree was similar to the phylogenetic trees mentioned above, which further verified the phylogenetic status of the 23 strains of the *Akkermansia* genus.

### Population structure

The population structure of the 23 *Akkermansia* genomes was investigated using STRUCTURE software based on the SNPs of core genes (Evanno et al. 2005). The result revealed that 23 *Akkermansia* strains were divided into 3 specific groups (Fig. 1b). The ATCC BAA-835, chimpanzee, elephant, horse, mouse, pig, reindeer, siamang, YL44, An78 and Urmite strains were clustered together, and most of genetic information of these strains was conserved. There was a little variation in the Urmite, UNK.MGS-1, 54_64, Phil8 and CAG:154, and strains ATCC BAA-835^T^, chimpanzee, elephant, horse, mouse, pig, reindeer, siamang, YL44 and An78 strains, showing high homology, which suggested that they evolved from a common ancestor. Phylogenetic analyses and dDDH values suggest that CAG:344 strain, KLE1605 and KLE1797 and KLE1798 should be classified as different species, and it’ll be more rational that the Urmite, UNK.MGS-1, 54_64, Phil8 and CAG:154, ATCC BAA-835, chimpanzee, elephant, horse, mouse, pig, reindeer, siamang, YL44 and An78 strains come from a common ancestor through the population structure analyses method.

### Local diversification of poly-clade genomes

To study the patterns of SNP distribution, SNP density was estimated throughout the 18 *A. muciniphila* genomes using a sliding window of 5 kb. In total, 533 SNPs regions throughout the genomes and SNPs were not evenly distributed among these regions (Database S1). Of the 533 regions, 9 regions had low density SNPs (less than 2.0 SNPs/kb), and 14 regions had high density SNPs (more than 75 SNPs/kb). The 9 regions with low density were related to Cell wall/membrane/envelope biogenesis. The 14 regions with high SNP density were related to metabolism and cell wall/membrane envelope biogenesis.

### Pan-genome analysis of *Akkermansia muciniphila*

To determine the core genes and strain-specific genes, 18 genomes of *A. muciniphila* were selected to analysis. Because some genomes were incomplete, a set of genes in their assemblies was missing. To determine the orthology genes, clustering was performed on the entire set of nucleotide sequences of all genes of each species instead of using sequence comparison against a reference strain. For the all-against-all comparison of all sequences, 95% was defined as the percentage sequence identity standard of nucleotide sequence.

**Fig. 2:**
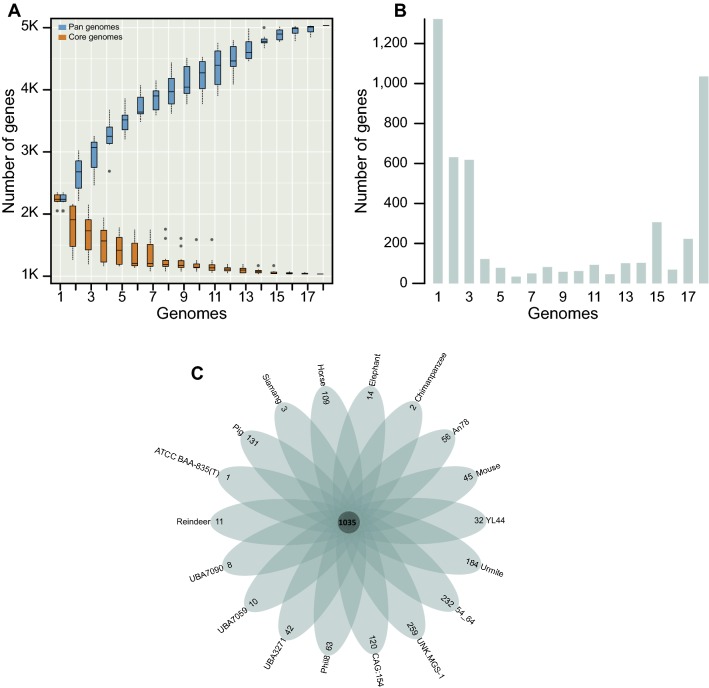
Pan-genome analyses of 18 *Akkermansia muciniphila* strains. **a** Pan-genome accumulation curves. The blue boxes denote the number of unique genes discovered with the sequential addition of new genomes. The orange boxes denote the number of core genes discovered with the sequential addition of new genomes. **b** Gene occurrence plot shows the core-genome and additional accessory genes of *Akkermansia muciniphila*. **c** Genomic diversity of 18 *Akkermansia muciniphila* strains. Each strain is shown as an oval. The number of core genomes is shown in the center. Overlapping regions show the genes conserved only within several strains. The numbers in non-overlapping portions show the number of strain-specific genes. The strain name is located beside the oval (color figure online)

Among the 18 genomes, 5033 unique protein-coding genes were identified in the pan-genome, corresponding to more than two-fold the average genes of the 18 genomes. The gene accumulation curve showed that the numbers of the core genome genes decreased continually with the addition of new strains, while the pan-genome showed an increasing trend (Fig. 2a). The change of both curves slowed down because the pan-genome of *Akkermanisa muciniphila* was in an open state, indicating that unique genes would be added along with the addition of new strains. The gene occurrence plot showed that a core-genome containing 1035 genes was present in all 18 *A. muciniphila* strains and that 1322 genes were strain-specific genes, and the plot also showed that the other genes were additional accessory genes mostly present in several genomes (Fig. 2b). The number of strain-specific genes in the 18 strains is shown in Fig. 2c. The remarkable difference of the specific genes between some strains suggested that these strains may have expanded in various habitats and that these strain-specific genes may have come from other species through lateral gene transfer (LGT). Furthermore, the strain-specific genes represented only a small number of strains, suggesting that the evolution of *A. muciniphila* was relatively conservative.

### Differentially enhanced functions in core and strain-specific genes

**Fig. 3:**
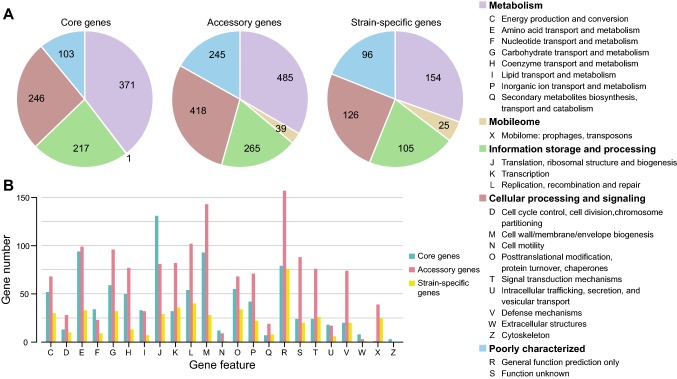
Differential distribution of COG functional categories in core and strain-specific genes. **a** Proportion of five classes of functional categories in core, accessory and strain-specific genes. **b** Functional categories in core, accessory and strain-specific genes

After obtaining core and strain-specific gene sequences, the distribution of their functional categories was compared using the Clusters of Orthologous Groups (COGs) database (Tatusov et al. 2003). However, the functional categories of only 82.4% (852/1035) of core genes, 35.1% (464/1322) of strain-specific genes and 49.7% (1331/2676) of accessory genes could be determined using the COGs database. Most of the genes did not exist in the COGs database, suggesting that *A. muciniphila* is poorly studied. The characteristics of relatively new species are scarce, thus suggesting the need to strengthen in-depth studies. This lack of in-depth knowledge may explain why functional categories could not be determined for many genes. The most abundant functions in the core genes of *A. muciniphila* were associated with metabolism (Fig. 3a). The overall proportion of genes related to metabolic functions was 39.6%, 30.4%, and 33.4% in the core genes, strain-specific genes and accessory genes, respectively. More specifically, translation, ribosomal structure and biogenesis (J), amino acid transport and metabolism (E), and cell wall/membrane/envelope biogenesis (M) were abundant in the core genes, suggesting that these genes were relatively conserved in *A. muciniphila*. The mobilome-related functions, such as prophage and transposase proteins (X), were more abundant in strain-specific genes than core genes (1 Mobilome-related prophage and transposon (X) in core genes; but 25 Mobilome-related prophage and transposon (X) in strain-specific genes). These results suggested that these strain-specific genes may have been transferred horizontally from other species or even from another genus and that the functions were not relatively important (Fig. 3b).

The number of the specific genes among the 18 genomes was 1322, ranging from 1 to 259 for each strain. The lowest number was encoded by *A. muciniphila* ATCC BAA-835^T^, and the highest number was identified in *Akkermansia* sp. UNK.MGS-1. Although a high number of strain-specific genes (approximately 65%) was not assigned to the COG categories, the other strain-specific genes fell into different functional categories. A higher proportion of strain-specific genes in most of the strains was assigned to the transcription (K), replication, recombination and repair (L), carbohydrate transport and metabolism (G), amino acid transport and metabolism (E), translation, ribosomal structure and biogenesis (J) and energy production and conversion (C) categories (Fig S3).

### Analyses of gene clusters and genomic islands of *Akkermansia*

The genomes of many microorganisms contain multiple biosynthetic gene clusters (BGCs) that code for production of secondary metabolites (Blin et al. 2017). These secondary metabolites play key roles in scientific research. The *A. muciniphila* ATCC BAA-835^T^ strain was selected as the reference to identify gene clusters using anti-SMASH 4.0 (Blin et al. 2017). Only 3 clusters responsible for the biosynthesis of secondary metabolites were identified as follows: 2 clusters belonged to terpenes; and 1 cluster belonged to arylpolyene. Moreover, the structures of the secondary metabolites coded by the 3 gene clusters could not be speculated. The locations of the 3 gene clusters on the *A. muciniphila* ATCC BAA-835^T^ reference genome were from 421,014 to 441,913, from 1,642,673 to 1,683,848 and from 1,745,606 to 1,766,541. The nucleotide sequence lengths of the 3 gene clusters were 20,899 bp, 41,175 bp and 20,935 bp, and the gene numbers of these clusters were 16, 26 and 25, respectively. The secondary metabolites may play a part in prebiotic functions, and the isolation, purification and structure identification of these secondary metabolites should be completed in future studies.

Genomic islands (GIs) are a major driver of genome evolution as they often provide adaptive traits that enhance the fitness of bacteria and archaea within a niche (Aminov 2011; Dobrindt et al. 2004). Eight genomic islands were identified in the *A. muciniphila* ATCC BAA-835^T^ genome sequences, and the gene numbers of all genomic islands were 5, 6, 8, 12, 7, 23, 5 and 8. The locations of these genomic islands are listed in Table S3. Compared with gene clusters, the genomic islands were smaller and included fewer genes. However, the functions of the genomic islands remain unknown. Analysis of all *Akkermansia* genomes revealed that only several strains had all 8 genomic islands mentioned above and that several strains only had a portion of the 8 genomic islands. These results suggested that the genes from genomic islands probably had a horizontal origin from other bacteria or archaea in different niches via different methods.

**Fig. 4:**
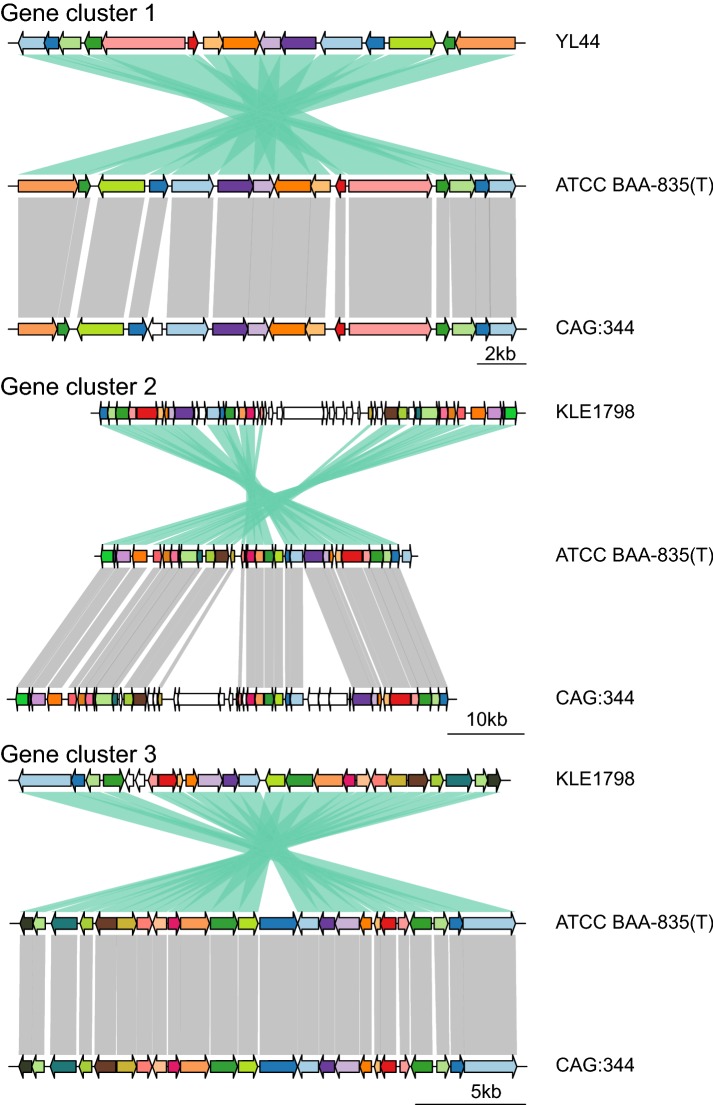
Comparison of biosynthetic gene clusters between *Akkermansia muciniphila* ATCC BAA-835^T^ and other strains of the *Akkermansia* genus. Regions of conserved synteny were marked with gray (+) and green (−) shadows. Different genes are shown by different color arrows, and genes with the same color are homologous to each other (color figure online)

Synteny analyses of the 3 gene clusters and the 8 genomic islands between *A. muciniphila* ATCC BAA-835^T^ and other strains of *Akkermansia* suggested that not all of the strains had these genes. The use of orthoMCL (Li et al. 2003) determined that all orthologous genes of the 23 *Akkermansia* strains studied in this article had 70% sequence identity of standard amino acid sequences. *A. muciniphila* ATCC BAA-835^T^ was used as a reference to identify the same gene clusters and genomic islands in other strains. The *A. glycaniphila* Pyt^T^ strain did not have similar gene clusters or genomic islands with the reference, which was potentially due to different evolution habitats. Furthermore, some clustered orthologous genes that were found in one strain had different positions, sequences, numbers, and orientation of the homologous genes among the strains, which may have been due to mutation, recombination and rearrangement events of these genes. The numbers and compositions of orthologous genes are listed in Table S4. The gene clusters were more conserved compared to genomic islands as shown in Table S5. Synteny maps comparing the gene clusters are shown in Fig. 4 and the genomic islands are shown in Figure S4.

### Analysis of Amuc_1100 cluster

**Fig. 5:**
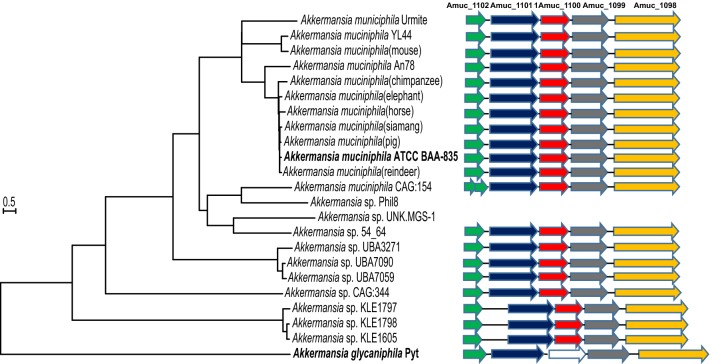
A gene cluster corresponding to locus tags from Amuc_1098 to Amuc_1102 in 23 *Akkermansia* strains. The colors represent the following genes: green represents Amuc_1102, blue represents Amuc_1101, red represents Amuc_1100, gray represents Amuc_1099 and yellow represents Amuc_1098. The white arrow indicates that no homologous gene of Amuc_1100 existed in the *Akkermansia glycaniphila* Pyt^T^ genome (color figure online)

Amuc_1100, a thermostable outer-membrane protein of *A. muciniphila* has been reported to play an important role to reduce fat mass and metabolic syndrome in mice with diet-induced obesity (Anhê and Marette 2017). Locus tags for Amuc_1098 to Amuc_1102 were clustered into a gene cluster. Amuc_1098 was predicted to encode a type II and type III secretion system protein, and Amuc_1101 was predicted to encode a cell division protein FtsA. The other 3 genes were annotated as hypothetical proteins (Ottman et al. 2017). *A. muciniphila*, whether live or pasteurized, and Amuc_1100 could decreased high cholesterol levels. The function of Amuc_1100 was related to formation of pili, thus could participate in the interaction between the bacterium and Toll-like receptor2 (TLR2) (Cani and de Vos 2017). Because of the important function of Amuc_1100 and strong candidate for future drug development, so we make further analyses of this cluster. For this cluster, gene order, length and orientation were conserved throughout the *Akkermansia* genus (Fig. 5), for the CAG:154 strain genome. Amuc_1102 was divided into 2 genes, and this cluster was not present in the Phil8 and UNK.MGS-1 strains, which may have been due to incomplete genome drafts. The distance between Amuc_1101 and Amuc_1102 was longer for the KLE1605, KLE1797 and KLE1798 strains compared to the other strains. The homologous genes for the Amuc_1100 cluster of *A. glycaniphila* Pyt^T^ were much longer. The homologous genes of Amuc_1100 in the *Akkermansia* genus were identified, except for the *A. glycaniphila* Pyt^T^ strain, suggesting that *A. glycaniphila* Pyt^T^ may not have the similarity function as other species in the *Akkermansia* genus.

### Genes related to mucin degradation

The analysis of *A. muciniphila* ATCC BAA-835^T^ genome predicts that over 61 (11%) of proteins are involved in the degradation of mucin (Belzer and de Vos 2012). Predicted function of these genes has been mentioned in the previous study performed by other groups (Derrien et al. 2010). Comparative analysis of the other strains and *A. muciniphila* ATCC BAA-835^T^, the distributions of these genes in different strains were displayed in Table S5. The results suggested that the strains which were more related to *A. muciniphila* ATCC BAA-835^T^ own more homologous genes related to mucin degradation.

## Discussion

Phylogenetic and DDH similarity analyses of 23 strains of the *Akkermansia* genus indicated that there are 4 species in this genus besides the 2 previously published *Akkermansia* species. Despite various habitats, the *A. muciniphila* strains were similar, suggesting that the evolution of *A. muciniphila* was relatively conservative. Population structure analysis supported the phylogenetic analysis results. Pan-genome analysis of 18 *A. muciniphila* genome sequences showed that the strain-specific genes were only a small proportion for each strain. The gene accumulation curve indicated that the pan-genome of *A. muciniphila* was in the open state and that it increased with the addition of new strains. Synteny analyses of gene clusters and genomic islands showed that only several strains had homologous sequences with *A. muciniphila* ATCC BAA-835^T^. The important thermostable outer-membrane protein, Amuc_1100, which is related to fat mass and metabolic syndrome, was not present in *Akkermasia glycaniphila* Pyt^T^. Therefore, the characteristics of the important gut probiotic, *Akkermasia,* remain unknown, and future efforts need to be developed for in-depth understanding and possible application of the probiotic.

## Electronic supplementary material

Below is the link to the electronic supplementary material.

Supplementary material 1 (XLSX 94 kb)

Supplementary material 2 (XLSX 12 kb)

Supplementary material 3 (XLSX 13 kb)

Supplementary material 4 (XLSX 9 kb)

Supplementary material 5 (XLSX 14 kb)

Supplementary material 6 (XLSX 15 kb)

Supplementary material 7 (DOCX 580 kb)
