# Supplementary material for: Comparative genomic and functional analysis of Akkermansia muciniphila and closely related species
Source: Genes Genomics. 2019 Aug 9;41(11):1253–64. doi: 10.1007/s13258-019-00855-1 (PMC6828834; doi:10.1007/s13258-019-00855-1)
Supplement: Supplementary file 7 — Supplementary material 7 (DOCX 580 kb) [file 13258_2019_855_MOESM7_ESM.docx]

**Comparative Genomic and Functional Analysis of *Akkermansia muciniphila* and Closely Related Species**

Juyuan Xing^2#^, Xiaobo Li^3#^, Yingjiao Sun^1^, Juanjuan Zhao^1^, Shaohua Miao^1^, Qin Xiong^1^, Yonggang Zhang^4^* & Guishan Zhang^1^*

^1^Key Laboratory of Microbial Resources Collection and Preservation, Ministry of Agriculture, Institute of Agricultural Resources and Regional Planning, Chinese Academy of Agricultural Sciences, Beijing, P. R. China

^2^Wuhan University of Technology, Wuhan, Hubei Province, P. R. China

^3^BGI Education Center, University of Chinese Academy of Sciences, Shenzhen, China

^4^Biology Institute, Qilu University of Technology (Shandong Academy of Sciences), Jinan, Shandong Province, P. R. China.

^#^co-first author, equal contribution.

**^*^Correspondence:**

Guishan Zhang, Key Laboratory of Microbial Resources Collection and Preservation, Ministry of Agriculture, Institute of Agricultural Resources and Regional Planning, Chinese Academy of Agricultural Sciences. No. 12 Zhongguancun South Street, Haidian District, Beijing 100081, P.R. China.

Yonggang Zhang, Biology Institute, Qilu University of Technology (Shandong Academy of Sciences), No. 19 Keyuan Road, Jinan 250014, Shandong Province, P. R. China.

E-mail: gszhang86@gmail.com; zhangygcq@163.com

List of author’s name and e-mail address:

Juyuan Xing junru1120102514@gmail.com

Xiaobo Li 815752398@qq.com

Yingjiao Sun 1153501575@qq.com

Juanjuan Zhao zhaojuanjuan3867@163.com

Shaohua Miao 1536115780@qq.com

Qin Xiong skyxiongqin@163.com

Yonggang Zhang zhangygcq@163.com

Guishan Zhang gszhang86@gmail.com

**Key words:** *Akkermansia muciniphila*; Comparative Genomic Analysis; Phylogenetic tree


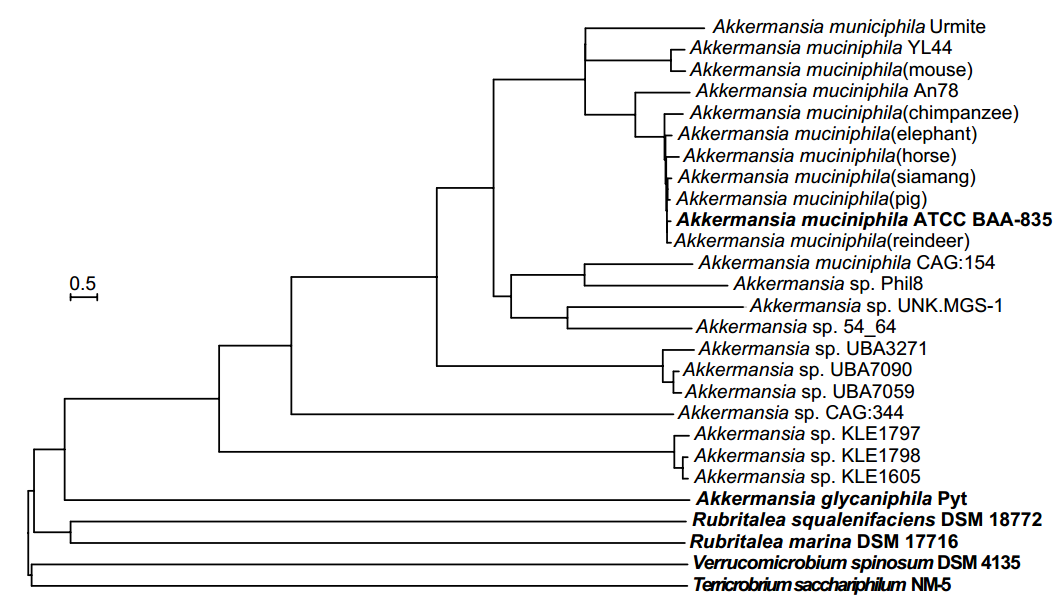


Figure S1. Phylogenetic tree of 23 *Akkermansia* genomes and 4 its closely related genomes of the phylum *Verrucomicrobia*. The phylogenetic tree was constructed using all amino acid sequences of each strains which studied in this article on the Web Sever of Composition Vector Tree Version 3 (CVTree3), and 6 was the K-tuple length. The phylogenetic tree was rooted by the out-group.


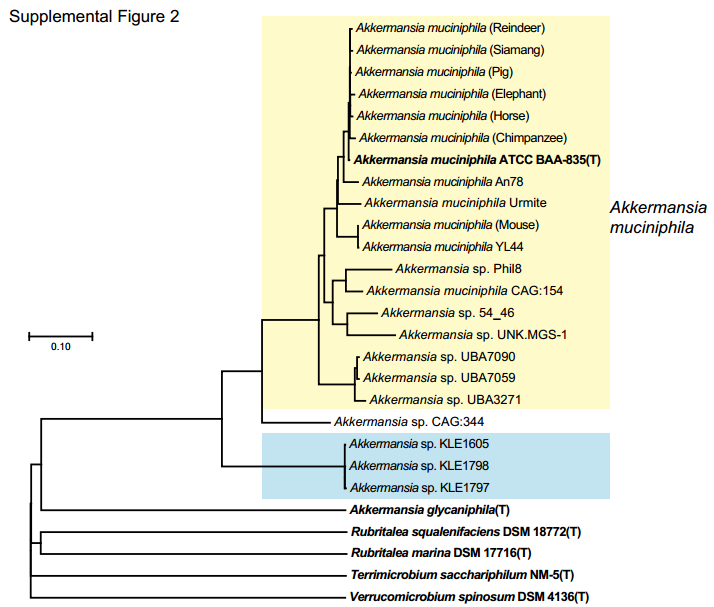


Figure S2. Phylogenetic tree of 23 *Akkermansia* genomes and 4 its closely related genomes of the phylum *Verrucomicrobia*. The phylogenetic tree was constructed using the matrix of Genome-to-Genome Distance (GGDs) of all genomes studies in this article with the software FastME. The phylogenetic tree was rooted by the out-group.


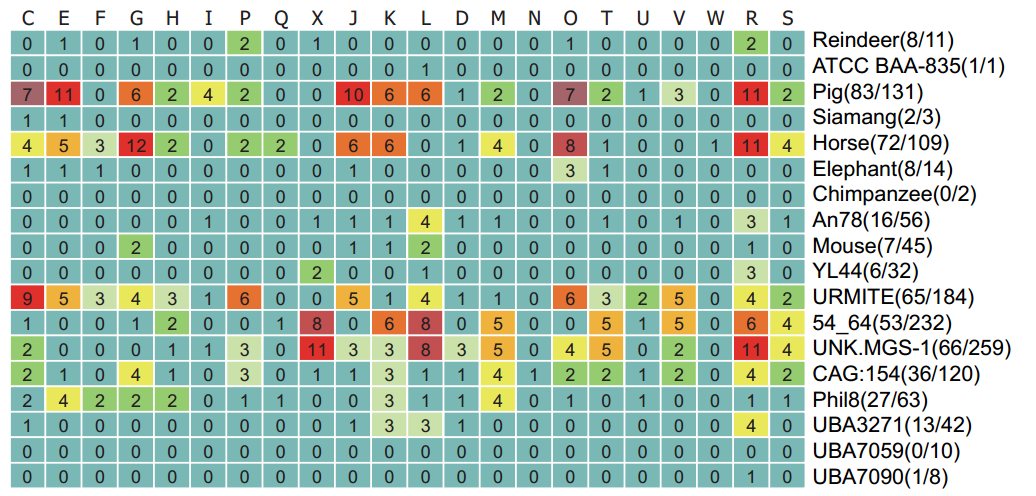


Figure S3. Functional classification of strain-specific genes in 18 *Akkermansia muciniphila* strains. The number in each square represents the COG assignment in each functional category. The annotated specific gene number in each functional category and the total specific gene number were listed behind *Akkermansia muciniphila* strain number.


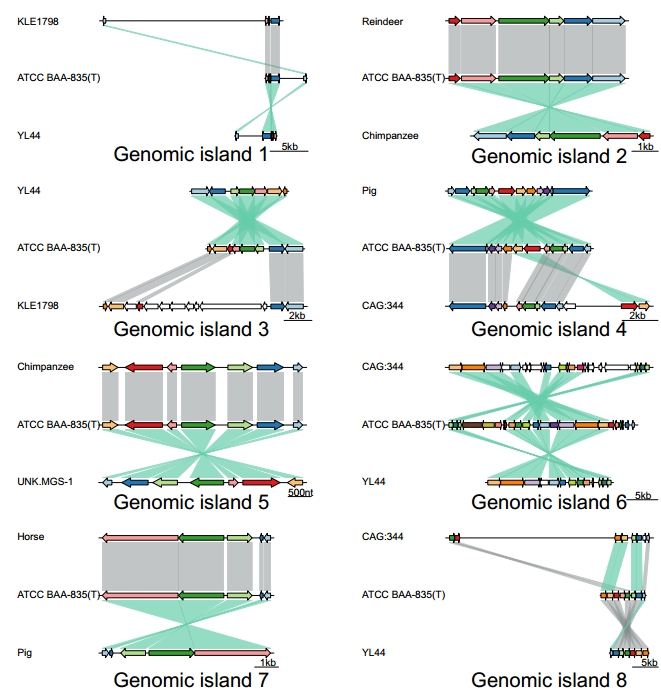


Figure S4. Comparison of biosynthetic genomic islands between *Akkermansia muciniphila* ATCC BAA-835^T^ and other strains of the *Akkermansia* genus. Regions of conserved synteny were marked with gray (+) and green (-) shadows. Different genes are shown by different color arrows, and genes with the same color are homologous to each other.
